# Transcriptome Analysis in Peripheral Blood of Humans Exposed to Environmental Carcinogens: A Promising New Biomarker in Environmental Health Studies

**DOI:** 10.1289/ehp.11401

**Published:** 2008-06-23

**Authors:** Danitsja M. van Leeuwen, Ralph W.H. Gottschalk, Greet Schoeters, Nicolas A. van Larebeke, Vera Nelen, Willy F. Baeyens, Jos C.S. Kleinjans, Joost H.M. van Delft

**Affiliations:** 1 Department of Health Risk Analysis and Toxicology, Maastricht University, Maastricht, the Netherlands; 2 Center of Expertise in Environmental Toxicology, Flemish Institute for Technological Research, Mol, Belgium; 3 Study Centre for Carcinogenesis and Primary Prevention of Cancer, Ghent University Hospital, Ghent, Belgium; 4 Provincial Institute of Hygiene, Antwerp, Belgium; 5 Laboratory of Analytical and Environmental Chemistry, Vrije Universiteit Brussel, Brussels, Belgium

**Keywords:** biomarker, biomonitoring, environmental carcinogens, human blood, transcriptomics

## Abstract

**Background:**

Human carcinogenesis is known to be initiated and/or promoted by exposure to chemicals that occur in the environment. Molecular cancer epidemiology is used to identify human environmental cancer risks by applying a range of effect biomarkers, which tend to be nonspecific and do not generate insights into underlying modes of action. Toxicogenomic technologies may improve on this by providing the opportunity to identify molecular biomarkers consisting of altered gene expression profiles.

**Objectives:**

The aim of the present study was to monitor the expression of selected genes in a random sample of adults in Flanders selected from specific regions with (presumably) different environmental burdens. Furthermore, associations of gene expression with blood and urinary measures of biomarkers of exposure, early phenotypic effects, and tumor markers were investigated.

**Results:**

Individual gene expression of cytochrome p450 1B1, activating transcription factor 4, mitogen-activated protein kinase 14, superoxide dismutase 2 (Mn), chemokine (C-X-C motif) lig-and 1 (melanoma growth stimulating activity, alpha), diacylglycerol *O*-acyltransferase homolog 2 (mouse), tigger transposable element derived 3, and PTEN-induced putative kinase1 were measured by means of quantitative polymerase chain reaction in peripheral blood cells of 398 individuals. After correction for the confounding effect of tobacco smoking, inhabitants of the Olen region showed the highest differences in gene expression levels compared with inhabitants from the Gent and fruit cultivation regions. Importantly, we observed multiple significant correlations of particular gene expressions with blood and urinary measures of various environmental carcinogens.

**Conclusions:**

Considering the observed significant differences between gene expression levels in inhabitants of various regions in Flanders and the associations of gene expression with blood or urinary measures of environmental carcinogens, we conclude that gene expression profiling appears promising as a tool for biological monitoring in relation to environmental exposures in humans.

Cancer is among the major causes of death in modern society (Boyle and Ferlay 2005). Carcinogenesis is initiated and/or promoted by exposure to chemicals with carcinogenic properties that occur in the human environment, for example, from ambient air, food, or lifestyle-related factors such as cigarette smoke (Czene et al. 2002; Higginson 1993; Le Marchand 2005; Luch 2005). Thus, in theory, a portion of cancer cases is preventable. Molecular epidemiology tools are used to identify human cancer risks posed by exposure to environmental carcinogens, thereby contributing to the scientific basis for environmental health policy measures. To this end, biomarkers are investigated that enable human risk assessment long before traceable, diagnostic health effects appear.

In monitoring environmental cancer risk among human populations, several markers of early biological effects [e.g., DNA damage markers such as micronuclei (MN) and DNA strand breaks] have been developed and applied over the last decades (Bonassi et al. 2005). However, these biomarkers tend to be nonspecific, raise questions about their sensitivity, and do not generate insights in underlying modes of action. Genomic technologies such as microarrays and quantitative polymerase chain reaction (PCR) provide the opportunity to explore altered expression of large numbers of genes simultaneously (Afshari et al. 1999; Gerhold et al. 2001; Lobenhofer et al. 2001; Lockhart and Winzeler 2000) and therefore may provide the opportunity to identify molecular biomarkers consisting of altered gene expression profiles representing environmental health risks. Studies on carcinogen-induced differential gene expression have been conducted in our laboratory previously. Besides experimental *in vitro* studies that were directed toward obtaining gene expression profiles from model carcinogens in human blood mono-nuclear cells (van Leeuwen et al. 2005), a concise human population of smoking-discordant monozygotic twin pairs has been investigated; genes were identified of which the expression significantly differed in smokers compared with their nonsmoking, genetically identical siblings (van Leeuwen et al. 2007). Furthermore, in a study of children from the Czech Republic, numerous gene expressions appeared relatively increased among children inhabiting a severely polluted area (van Leeuwen et al. 2006).

From these studies, eight genes have been identified as promising biomarkers for environmental carcinogenesis. They encompass genes of which the expression differed significantly between carcinogen-exposed and non-exposed individuals, in addition to genes that correlated significantly with an established biomarker of early biological effect (i.e., MN frequencies) (van Leeuwen et al. 2006, 2007). The aim of the present study was to monitor the expression of this set of genes in humans inhabiting specific regions in Flanders and to associate these with blood and urinary measures of established biomarkers of exposure and early biological effect. We measured the expression levels of these eight key genes—cytochrome P450 1B1 (*CYP1B1*)*,* activating transcription factor 4 (*ATF4*)*,* mitogen-activated protein kinase 14 (*MAPK14*)*,* superoxide dismutase 2 (Mn) (*SOD2*)*,* chemokine (C-X-C motif) ligand 1 (melanoma growth stimulating activity, alpha) (*CXCL1*)*,* diacylglycerol *O*-acyltransferase homolog 2 (mouse) (*DGAT2*)*,* tigger transposable element derived 3 (*TIGD3*), and PTEN-induced putative kinase-1 (*PINK1*) (van Leeuwen et al. 2006, 2007) (Table 1)—in peripheral blood cells by means of quantitative PCR. Furthermore, we explored associations with blood and urinary measures of biomarkers of exposure to certain environmental carcinogens, biomarkers of early biological effect (DNA strand breaks and MN frequencies), and serum levels of several tumor markers. The present study was conducted as part of the Flanders Environment and Health Study (FLEHS; Flemish Centre of Expertise on Environment and Health 2007). FLEHS is a multicenter program with the goal of researching the impact of the environment on human health across the general Flanders population.

## Materials and Methods

### Study population

The study population consisted of 398 subjects from eight different regions of residence in Flanders, Belgium (Table 2). Participants were recruited within several communities or sectors of communities in each of the eight regions of interest, based on random sampling. Inclusion criteria were age 50–65 years, living in the region > 5 years, and being able to complete questionnaires in Dutch. Prior to blood collection, informed consent was obtained from all individuals. Study protocols were approved by the Institutional Review Board/Ethical Committee of Antwerp University. Participants completed a questionnaire covering age, sex, and smoking habits, among other items, and they donated a blood and urine sample for measurement of the biomarkers. The questionnaire was based partly on a questionnaire used in the pilot phase of the FLEHS project (Staessen et al. 2001) and supplemented with specific questions concerning the current study.

### Gene expression analysis

For gene expression analyses, blood samples of 2.5 mL were collected from each subject into PAXgene Blood RNA vacutainer tubes (PreAnalytix; Qiagen, Hilden, Germany), a system that accounts for immediate *ex vivo* preservation of blood RNA. Total RNA was isolated and purified using the PAXgene Blood RNA kit (PreAnalytix) according to the manufacturer’s instructions. cDNA was synthesized from 2 μg total RNA using the BioRad iScript cDNA synthesis kit (Bio-Rad Laboratories, Hercules, CA, USA) according to the manufacturer’s instructions. Aliquots were used for quantitative PCR on the BioRad MyiQ iCycler Single Color quantitative detection system using iQ SYBR Green Supermix (both from Bio-Rad) according to the manufacturer’s instructions. Reactions were initiated for 3 min at 95°C, followed by 40 cycles of 15 sec at 95°C and 45 sec at 60°C. After each run, we performed a melting curve analysis starting at 60°C with stepwise temperature elevations of 0.5°C every 10 sec to check for nonspecific products. We included β-actin (*ACTB*) and cyclophillin A (*PPIA*) as reference genes (internal controls). Primers were as follows: *ACTB*, 5′-CCTGGCACCCAGCA-CAAT-3′ (forward) and 5′-GCCGATCCA-CACGGAGTACT-3′ (reverse); and *PPIA,* 5′-T T C C T G C T T T C A C A G A A T T ATTCC-3′ (forward) and 5′-GCCACCAG-TGCCATTATGG-3′ (reverse). These genes perform best in terms of most stable expression and best resemblance to microarray-derived results in our previous analyses (data not shown). All reactions were performed in duplicate. In each run, negative controls (not containing template) and positive controls (a dilution series of a pooled sample, consisting of cDNA reverse-transcribed from total RNA of 20 randomly selected subjects) were included to estimate PCR efficiency. Primer sequences are shown in Table 1.

### Exposure analysis

We measured whole blood, serum, or urine levels of multiple environmental carcinogens or their metabolites by various methods: heavy metals (cadmium and lead) in whole blood as described by Schroijen et al. (2008); dioxins and furans in serum as described by Van Wouwe et al. (2004); *p,p*′-dichlorodiphenyldichloro-ethylene (*p,p*′-DDE) and non–dioxin-like polychlorinated biphenyls (PCBs) in serum as described by Covaci et al. (2002) and Gomara et al. (2002); 1-OH-pyrene (a metabolite of polycyclic aromatic hydrocarbons) and *t,t*-muconic acid (*t,t*-MA; a metabolite of benzene) in urine as described by Angerer and Schaller (1997, 1998). Smoking status was derived from questionnaires instead of cotinine measurements.

### Measurement of early biological effect and tumor markers

We evaluated the induction of DNA strand breaks as a measure for DNA damage using the alkaline COMET assay as described by Singh et al. (1988). We analyzed the slides (200 cells per individual) using an image analysis system from Metasystems (Altslussheim, Germany). Median percentages of DNA migration in the tail areas were determined and used as a measure of DNA damage. As a positive control, one slide with nuclei from deep-frozen whole blood was added to each electrophoresis chamber. DNA migration in positive controls had to be > 30%. To investigate the MN frequencies, we performed the cytokinesis-block micronucleus assay on whole blood cultures using standard procedures according to Fenech (2000). For each individual, we evaluated 1,000–2,200 cells for the presence of micronuclei using the Metafer automatic program (Metasystems). We measured 8-hydroxydeoxyguanosine (8-OH-dG) in urine by means of ELISA using the competitive immunosorbent assay (Gentaur, Brussels, Belgium) according to the manufacturer’s instructions. Serum protein levels of the tumor marker p53 were analyzed using the enzyme immunometric assay and Titerzyme EIA p53 (Assay Designs, Ann Arbor, MI, USA). Carcino-embryonic antigen (CEA) and prostate-specific antigen (PSA) levels were measured using a solid-phase chemiluminescent immunometric assay and Immulite 2000 (DPC, Los Angeles, CA, USA). Samples were analyzed for PSA within 24 hr after collection.

### Data analysis

Ct values [concentration of DNA molecules (in moles) multiplied by time] per subject and per gene were normalized by subtraction of the mean Ct value of *ACTB* and *PPIA*. Subsequently, we calculated Δ ΔCt values per subject and per gene relative to the normalized pooled reference sample within each quantitative PCR run using the following formula:


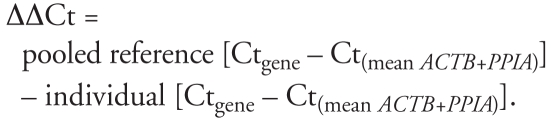


This generated one expression value per gene per individual study participant. All gene expression data are reported as ΔΔCt values on log (base = 2) scale (Livak and Schmittgen 2001) and presented as average group values. Next to gene expression for the eight individual genes, an integrative expression value was calculated as the mean of the eight individual genes.

We performed statistical testing using SPSS 14.0 (SPPS Inc., Chicago, IL, USA). We used one-way analysis of variance (ANOVA) with post hoc Bonferroni correction to test for significance of differences in gene expression between groups of individuals inhabiting different regions and between groups of individuals with different smoking status (current vs. former, current vs. never-smoker, and former vs. never-smoker) and other variables, as well as blood and urinary measures of exposure and effect biomarkers. For examination of intervariable correlations at the level of all individuals (no division based on region of inhabitance), we applied Pearson correlation analysis. *p*-Values <0.05 are considered statistically significant.

## Results

**Figure 1:**
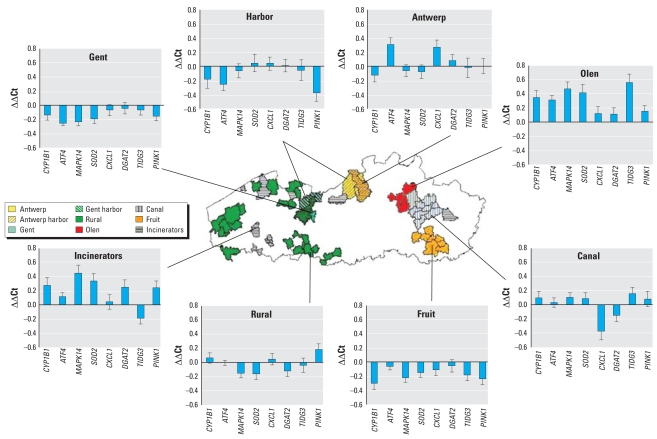
Gene expression (mean ± SE) per region, relative to the total population averages, for all nonsmokers (former and never-smokers). Fruit, fruit cultivation region.

Tables 3–5 describe the characteristics of the study population according to the region of inhabitance and of the population as a whole. For seven individuals (1.8%), the smoking status was unknown; therefore, these were excluded from the analyses. Across regions, participant groups did not differ significantly with respect to age, sex, and smoking status. Statistical analysis of differences in gene expression between current, former, and never-smokers revealed *CYP1B1* expression levels to be significantly different between current and former smokers (*p* = 0.029) and between current and never-smokers (*p* < 0.001). Because of the apparent confounding effect of smoking, we further investigated gene expression in non-smokers (i.e., never and former smokers only), changing the size of the total population to 319 individuals. Per region, at least 29 individuals remained in the analyses; therefore, we consider the populations still of adequate size in terms of power. A map of Flanders with bar charts of the average gene expressions among habitants per region is shown in Figure 1. Compared with the total population average, subjects with the most distinct gene expression profiles live in Olen (expressions well above the population average) as well as in the fruit cultivation region and in Gent (both with expressions well below the population average). In a one-way ANOVA analysis with a post hoc Bonferroni test, all genes appeared to significantly differ in expression between inhabitants from two or more regions (*p* < 0.003), except for *DGAT2* (*p* = 0.06). Based on the individual gene expression as well as the mean/sum of all gene expressions, inhabitants from Olen show the most significant differences compared with subjects living in the fruit cultivation region (Fruit) and Gent (*p* < 0.001). We performed Pearson correlation analyses to investigate associations between individual gene expression (i.e., gene expression values per study participant regardless of region of inhabitance) and blood and urinary measures of biomarkers of exposure, early biological effect, and tumor markers. These analyses were carried out for the total nonsmoking population (composed of never and former smokers) or separately for female and male participants. Significant correlations between gene expression and blood and urinary measures of biomarkers of exposure are presented in Table 6 (all currently nonsmoking individuals, females only, and males only).

## Discussion

The FLEHS project was initiated by the Flemish government in 2001, with the plan to use the forthcoming study results in environmental risk assessment and environmental health policy making. Flanders typically comprises a range of environmental burdens such as urban areas, regions with dense traffic, intensive agriculture, and industry. In the present cross-sectional study, we investigated the expression of eight key genes in peripheral blood sampled from the adult Flanders population by means of quantitative PCR.

Except for *DGAT2*, all gene expression levels differed significantly between current nonsmoking (i.e., never-smoking and formerly smoking) inhabitants from two or more regions. Based on individual gene expression as well as the mean of all gene expressions, the most deviating region is Olen, because its inhabitants returned the most significant differences compared with inhabitants of the other regions, in particular Harbor, Fruit, Gent, and Rural (Figure 1).

Many significant correlations of gene expression with endogenous levels of relevant environmental carcinogens were observed among all currently nonsmoking individuals; Cd in blood or urine, PCBs, dioxins and furans, hexachlorobenzene (HCB), *p,p*′-DDE, *t,t*-MA, and 1-OH-pyrene (Table 6). The majority of these associations have not yet been described in literature, whereas reported biological functions of these gene expressions have been linked to carcinogenesis. All but one of these correlations were positive. The only negative correlation was found between *CYP1B1* expression levels and urinary levels of *t,t*-MA. Up to now, *CYP1B1* gene expression *in vivo* has not been reported to be influenced by benzene or its metabolites such as *t,t*-MA. *CYP1B1* gene expression is known to be inducible by dioxin and PAH. This was not demonstrated in this population, possibly because of heterogeneity of the general population for blood levels of this environmental carcinogen. *SOD2* expression correlated significantly with blood or urine measures of three exposure markers: PCBs, *p,p*′-DDE, and Cd in urine. Acknowledging *SOD2* for its function in oxidant scavenging, these correlations indicate elevated oxidative stress as a result from these exposures. Although this association has been reported extensively for Cd exposure (Bertin and Averbeck 2006), it has not for exposure to PCBs and DDT or DDE, although these compounds are metabolized through oxidative processes. *ATF4* expression levels showed a significant correlation with urinary levels of 1-OH-pyrene, a metabolite well known for representing PAH exposure. Although not previously reported, this might indicate that PAH exposure influences transcription. Furthermore, *ATF4* expression correlated with DNA strand breaks, expressed as COMET P90. *ATF4* is also known as *CREB2*, a member of CREB family, which is known as a key regulator in the control of cellular gene expression, regulating cell cycle and growth factor genes of which aberrant expression is observed in certain cancers (Cheng et al. 2007). *MAPK14* gene expression returned correlations with most of the environmental carcinogens; PCBs, HCB, *p,p*′-DDE, and Cd in blood as well as in urine. Upon environmental carcinogenic exposure, reported for PCB-47, *MAPK14* functions as a mediator of *COX2* gene expression (Bezdecny et al. 2007). In turn, *COX2* expression is known to be deregulated in certain tumors. Considering the substrates of this MAP kinase, ATF2, MEF2C, MAX, CDC25B, and p53, it is suggested to be associated with stress-related transcription and cell cycle regulation, as well as genotoxic stress response (Cuenda and Rousseau 2007). MAPK is also known for its extensive role in cell differentiation and proliferation, as well as its involvement in the SOS-Ras-Raf-MAPK cascade, which plays a central role in acquired growth signal autonomy, considered a key event on the route of cell normalcy to malignancy, and at other levels of the carcinogenic process (Cuenda and Rousseau 2007; Hanahan and Weinberg 2000). *PINK1* expression correlated significantly with endogenous levels of PCBs and *p,p*′-DDE. *PINK1* is a PTEN-induced putative kinase. PTEN is a tumor suppressor, functioning as an inhibitor of the AKT/PKB signaling pathway, and is mutated in a large number of cancers (Kim and Mak 2006). *CXCL1* is a chemokine whose gene expression was found in the present study to correlate significantly with blood measures of dioxins and furans. It has been associated with tumor growth and metastasis (Wang et al. 1998). *TIGD3* expression, a gene encoding a DNA-transposable element, correlated significantly with the blood or urinary measures of PCBs and with 1-OH-pyrene in urine. *TIGD3* expression also appeared to correlate with serum levels of p53. Furthermore, a positive significant correlation was found between dioxins/furans and *DGAT2* expression, a gene involved in triglyceride synthesis. The functions of these two genes as described in literature, however, do not indicate a relationship with environmental carcinogenesis.

When we assessed the influence of sex on the correlations of gene expressions and bio-markers of exposure, early effect, or tumor markers, we observed substantial differences between females and males. The correlation profile, as observed in males only, reflects the total population profile more than that of females only. This suggested unique correlation profile in females includes significant associations of gene expression with mostly biomarkers of early effect, such as *SOD2* expression correlating with the number of cells with MN, the number of MN per 1,000 binucleated cells, and the serum level of CEA. *CYP1B1* expression correlated with CEA levels. In addition, *DGAT2* expression correlated significantly with blood measures of Pb and median COMET values. Differences in kinetics, dynamics, and biotransformation of xenobiotics between the sexes (Beierle et al. 1999; Schwartz 2003) may explain the differences observed in the present study. These observations warrant more in-depth analysis of this effect in the future.

In general, the contributions of MAPK, CREB, and PTEN to the cell circuitry involved in carcinogenesis are also comprehensively summarized by Hanahan and Weinberg (2000) in their review on the essential alterations in cell physiology that establish malignant growth. Furthermore, observed effects on *CXCL1* may be linked with the process of environmental carcinogenesis. These gene expression markers appear promising in their qualitative use because of their biologically relevant modes of action and consequential broadening of the insights into cancer risks due to environmental carcinogen exposure. We therefore suggest that the observed dose–response relationships between these modified gene expressions and well-known environmental carcinogens as present in the Flemish population deepen our understanding in environmental cancer risks and clearly present the value of gene expression analysis as a tool for biological monitoring purposes.

In a few previous studies, gene expression has also been monitored in peripheral blood from human populations. Wu et al. (2003) described gene expression profiling in a Taiwanese population exposed to arsenic pollution through drinking water. They found differential expression between groups with low-, intermediate-, and high-arsenic exposure, in particular, inflammation-related genes showing up-regulation with increasing exposure. In addition, gene expression profiling has been used to examine the risks of occupational exposures to benzene and metal fumes (Forrest et al. 2005; Wang et al. 2005). However, these studies did not include as many study subjects as the present study.

## Conclusions

In this large cross-sectional study, we have demonstrated the potential of gene expression analysis for monitoring in relation to environmental carcinogenic exposures in humans. We found that a gene expression profile differed significantly between human populations according to their environmental exposure. Many correlations between gene expression and blood or urinary measures of biomarkers of exposure to environmental carcinogens were observed. Furthermore, we found evidence for the contribution of xenobiotic exposure to environmental carcinogenesis at the molecular level in terms of impact on the expression of genes related to metabolism, stress response, signaling pathways, and tumorigenesis. We therefore conclude that gene expression profiling appears promising for application to the analysis of environmental health risks. This should be considered with respect to the increased human biomonitoring activities as foreseen under the European Environment Action Programme (European Commission 2008).

## Figures and Tables

**Table 1:** Overview of genes monitored for expression in blood cells.

Gene name (abbreviation)^a^	GenBank accession no.^a^	Biological summary^a^	Primers
Cytochrome P450 1B1 (*CYP1B1*)	NM_000104	Catalysis of many reactions involved in drug and xenobiotic metabolism (e.g., metabolism of procarcinogens)	5′-AGTGCAGGCAGAATTGGATCA-3′ (forward) 5′-GCGCATGGCTTCATAAAGGA-3′ (reverse)
Activating transcription factor 4 (*ATF4*)	NM_001675	Encodes a transcription factor that belongs to a family of DNA-binding proteins, including the AP-1 and CREB families	5′-CTCCAGCGACAAGGCTAAGG-3′ (forward) 5′GTTGTTGGAGGGACTGACCAA-3′ (reverse)
Superoxide dismutase 2 (*SOD2*)	NM_000636	Associated with oxidative stress; converts superoxide to hydrogen peroxide and diatomic oxygen	5′-ATCAGGATCCACTGCAAGGAA-3′ (forward) 5′-CGTGCTCCCACACATCAATC-3′ (reverse)
Mitogen-activated protein kinase 14 (*MAPK14*)	NM_001315	Activated by various environmental stressors and proinflammatory cytokines; integration point for multiple biochemical signals and involved in a wide variety of cellular processes such as proliferation, differentiation, transcription regulation, and development	5′-TGAAGACTGTGAGCTGAAGATTCTG-3′ (forward) 5′ CCACGTAGCCTGTCATTTCATC-3′ (reverse)
Chemokine (C-X-C motif) ligand 1 (melanoma growth stimulating activity, alpha) (*CXCL1*)	NM_001511	Regulates cell trafficking of various types of leukocytes and has a role in development, homeostasis, and function of the immune system	5′-CCACTGCGCCCAAACC-3′ (forward) 5′-GCAGGATTGAGGCAAGCTTT-3′ (reverse)
PTEN-induced putative kinase-1 (*PINK1*)	NM_032409	Encodes a serine/threonine protein kinase that localizes to mitochondria; it is thought to protect cells from stress-induced mitochondrial dysfunction	5′-AGCAGTCACTTACAGAAAATCCAAGA-3′ (forward) 5′-GGTGAAGGCGCGGAGAA-3′ (reverse)
Diacylglycerol *O*-acyltransferase homolog 2 (mouse) (*DGAT2*)	NM_032564	Responsible for triglyceride synthesis	5′-GCACAGAGGCCACAGAAGTG-3′ (forward) 5′-CCCTCAACACAGGCATTCG-3′ (reverse)
Tigger transposable element derived 3 (*TIGD3*)	NM_145719	Belongs to the tigger subfamily of the pogo superfamily of DNA-mediated transposons in humans; exact function of gene is not known	5′-GTGCTGGAACTCCTGGATGAG-3′ (forward) 5′-TTGCAGATGCGCGAGATCT-3′ (reverse)

a National Center for Biotechnology Information (2008).

**Table 2:** Environmental characteristics of the study regions.

Region	Population (no.)	Description
Antwerp	404,241	City and suburbs with exclusion of harbor area
Harbor	130,064	Harbor areas of Antwerp and Gent combined (petrochemical and steel industries)
Fruit	95,829	Eight municipalities with > 10 ha/km^2^ apple and pear cultivation
Olen	68,068	Influenced by the presence of a nonferro industrial company
Gent	213,025	City and suburbs with exclusion of harbor area
Incinerators	56,405	Surroundings of 11 incinerators; municipalities with their center < 6 km from an incinerator (or < 12 km northeast of incinerator)
Rural	153,770	Twenty-four municipalities with population density < 250 persons/km^2^; no registered emission source; > 5% industry and no highway in the territory
Canal	64,763	Presence of six chemical companies (BP-Chembel, Exxon Mobil, Dow Chemical, Borealis, T.C. Ham, Tessenderlo Chemie-LVM)

Fruit, fruit cultivation region.

**Table 3:** Characteristics of the study population by region of residence.

Characteristic	Antwerp	Harbor	Fruit	Olen	Gent	Incinerator	Rural	Canal	Total
Subjects (no.)	50	41	35	39	72	50	76	35	398
Age (years)	58.6 ± 0.6	57.1 ± 0.7	57.0 ± 0.7	58.9 ± 0.7	58.9 ± 0.6	59.0 ± 0.6	59.3 ± 0.5	60.1 ± 0.6	59.2 ± 0.2
Sex [no. (%)]									
Males	30 (60)	20 (48.8)	19 (54)	17 (44)	38 (53)	25 (50)	42 (55.3)	16 (46)	207 (52)
Females	20 (40)	21 (51.2)	16 (46)	22 (56)	34 (47)	25 (50)	34 (44.7)	19 (54)	191 (48)
Smokers [no. (%)]									
Nonsmokers	19 (38)	19 (46.3)	19 (54.3)	17 (43.6)	38 (52.8)	24 (48)	43 (56.5)	17 (48.6)	196 (49.2)
Former	16 (32)	11 (26.8)	12 (34.3)	14 (35.9)	23 (31.9)	15 (30)	20 (26.3)	12 (34.3)	123 (30.9)
Current	15 (30)	6 (14.6)	4 (11.4)	7 (17.9)	11 (15.3)	11 (22)	12 (15.8)	6 (17.1)	72 (18.1)
Unknown	0 (0)	5 (12.2)	0 (0)	1 (2.6)	0 (0)	0 (0)	1 (1.3)	0 (0)	7 (1.8)

Fruit, fruit cultivation region. Values shown for age are mean ± SE.

**Table 4:** Blood or urine concentrations [mean (range)] of environmental pollutants by region of residence.

	Antwerp	Harbor	Fruit	Olen	Gent	Incinerators	Rural	Canal	Total
Cd (urine)^a^	0.73 (0.2–2.2)	0.83 (0.3–2.4)	0.72 (0.3–1.9)	0.86 (0.3–2.1)	0.61 (0.2–2.2)	0.82 (0.2–6.5)	0.61 (0.1–1.7)	0.78 (0.3–1.7)	0.72 (0.1–6.5)
Cd (blood)^b^	0.61 (0.1–1.7)	0.68 (0.2–1.6)	0.50 (0.1–1.8)	0.72 (0.2–3.0)	0.59 (0.1–3.4)	0.80 (0.2–1.8)	0.61 (0.1–2.4)	0.77 (0.3–2.0)	0.65 (0.1–3.4)
Pb (blood)^b^	44.60 (10.7–181.5)	38.41 (12.7–74.1)	39.04 (2.5–129.6)	43.58 (21.0–96.8)	45.24 (15.7–106.9)	46.07 (9.3–99.6)	51.19 (18.1–133.2)	37.78 (12.0–106.6)	44.41 (2.5–181.5)
1-OH-pyrene^a^	0.34 (0.02–2.3)	0.27 (0.01–2.2)	0.37 (0.02–2.0)	0.32 (0.02–1.8)	0.20 (0.01–1.2)	0.26 (0.01–1.8)	0.24 (0.01–1.3)	0.22 (0.01–1.0)	0.27 (0.01–2.3)
HCB^c^	71.19 (22.9–189.0)	63.00 (13.7–157.3)	62.18 (12.6–125.8)	82.74 (30.1–177.2)	63.52 (20.7–216.7)	74.40 (14.6–242.7)	73.95 (20.3–242.6)	74.71 (27.5–188.7)	70.62 (12.6–242.7)
*t,t*-MA^a^	0.16 (0.01–0.59)	0.12 (0.01–0.50)	0.16 (0.01–0.65)	0.14 (0.01–0.63)	0.14 (0.01–0.58)	0.12 (0.01–0.61)	0.13 (0.01–1.34)	0.09 (0.01–0.36)	0.13 (0.01–1.34)
PCBs^d^	382.4 (183.1–875.4)	363.2 (62.1–600.0)	368.9 (67.4–1061.6)	445.8 (126.5–919.3)	388.9 (152.4–949.5)	378.7 (42.3–710.7)	391.6 (75.5–765.4)	407.1 (211.5–1317.6)	390.4 (42.3–1317.6)
*p,p*′-DDE^c^	692.7 (11.4–3014.2)	527.2 (1.8–1926.2)	1189.2 (1.8–16967.0)	1444.3 (126.7–7575.8)	600.3 (61.8–4458.6)	813.1 (1.1–5208.4)	905.1 (30.8–3846.2)	1446.7 (89.0–8614.9)	900.8 (1.1–16967.0)
Dioxins^e^	28.85 (4.2–64.9)	17.68 (3.5–51.9)	41.67 (7.3–69.2)	16.84 (4.2–70.9)	20.08 (4.2–103.4)	20.65 (4.4–44.3)	25.80 (3.6–114.8)	19.58 (3.5–52.2)	23.26 (3.5–114.8)

Abbreviations: Fruit, fruit cultivation region; HCB, hexachlorobenzene. Individuals for whom smoking status was not known or reported are not included.

a mg/g creatinine.

b μg/L.

c ng/g fat.

d Sum of PCBs 138, 153, and 180; expressed as ng/g fat in serum.

e pg TEQ/gr fat in serum.

**Table 5:** Measurements [mean (range)] of early biological effect markers and tumor markers by region of residence.

	Antwerp	Harbor	Fruit	Olen	Gent	Incinerator	Rural	Canal	Total
Cells with MN (no.)	8.48 (1.8–20.0)	5.54 (0.8–15.2)	7.72 (0.3–14.5)	6.70 (1.5–16.4)	7.02 (0.9–18.3)	9.41 (0.0–35.9)	8.71 (1.0–22.7)	7.62 (1.8–28.8)	7.76 (0.0–35.9)
MN ‰^a^	9.41 (1.9–21.9)	5.99 (0.8–15.9)	8.45 (3.0–15.4)	7.23 (1.5–18.6)	7.75 (0.9–20.4)	10.57 (0.0–38.9)	9.14 (1.0–28.2)	8.84 (2.3–45.9)	8.63 (0.0–45.9)
8-OH-dG^b^	15.37 (5.1–32.8)	14.93 (5.9–23.0)	17.21 (7.3–32.4)	15.15 (5.7–40.3)	15.76 (7.2–29.6)	18.87 (3.7–57.2)	15.02 (3.7–42.2)	15.79 (7.9–24.5)	15.95 (3.7–57.2)
COMET (P90)^c^	8.90 (5.4–15.5)	12.97 (6.7–21.7)	10.06 (6.6–13.6)	8.82 (5.0–13.5)	8.43 (5.4–11.5)	8.06 (3.7–13.7)	8.63 (5.4–12.0)	8.58 (5.9–12.1)	8.82 (3.7–21.7)
COMET (median)^c^	1.75 (0.1–5.5)	3.85 (0.4–7.5)	2.83 (1.2–4.3)	1.65 (0.0–4.3)	2.33 (0.5–7.6)	1.79 (0.0–3.5)	2.29 (0.4–4.5)	1.95 (0.1–4.4)	2.14 (0.0–7.6)
PSA^d^	1.48 (0.3–4.9)	1.47 (0.3–3.9)	1.12 (0.3–4.8)	1.27 (0.2–4.1)	2.06 (0.3–27.1)	0.95 (0.2–4.4)	1.20 (0.0–3.8)	1.32 (0.4–4.0)	1.40 (0.0–27.1)
CEA^d^	2.65 (0.4–22.2)	1.96 (0.4–4.8)	2.03 (0.3–5.6)	2.45 (0.4–8.7)	2.37 (0.5–15.5)	2.66 (0.6–13.8)	2.61 (0.6–14.0)	1.63 (0.7–4.0)	2.39 (0.3–22.2)
p53^e^	41.1 (0.01–748.0)	176.6 (0.01–1378.0)	84.5 (0.01–804.0)	71.6 (0.1–1396.0)	86.9 (0.01–1531.0)	71.9 (0.01–1327.0)	95.9 (0.01–1620.0)	35.9 (0.01–507.0)	76.6 (0.01–1620.0)

Fruit, fruit cultivation region. Individuals for whom smoking status was not known or reported are not included.

a Number of micronuclei per 1,000 binucleated cells.

b μg/g creatinine.

c COMET was not measured in all study participants, but it was measured in at least 10 individuals per region; COMET P90 and COMET median in % DNA in the comet tail.

d ng/mL.

e pg/mL.

**Table 6:** Correlation coefficients (CCs) of the correlations of any of the gene expressions with blood or urinary measures of biomarkers of exposure, markers of early biological effect, or tumor markers, regardless of regions of residence, among current nonsmokers (never-smokers and former smokers combined) for all individuals, female participants only, and male participants only.

	*CYP1B1*	*ATF4*	*MAPK14*	*SOD2*	*CXCL1*	*DGAT2*	*TIGD3*	*PINK1*		*CYP1B1*	*ATF4*	*MAPK14*	*SOD2*	*CXCL1*	*DGAT2*	*TIGD3*	*PINK1*
All individuals									Females only								
Dioxins									1-OH-pyrene								
CC	−0.021	−0.058	−0.008	−0.006	0.136	0.119	−0.069	0.068	CC	−0.033	0.147	−0.069	−0.066	0.001	−0.144	0.146	−0.041
*p*-Value	0.723	0.323	0.898	0.918	0.021*	0.043*	0.242	0.248	*p*-Value	0.682	0.065	0.387	0.411	0.986	0.071	0.066	0.605
PCBs									COMET (median)								
CC	0.021	0.053	0.127	0.145	0.076	0.089	0.143	0.125	CC	0.150	0.041	0.085	0.138	0.124	0.255	0.106	0.134
*p*-Value	0.704	0.345	0.023*	0.010*	0.177	0.111	0.010*	0.026*	*p*-Value	0.106	0.662	0.358	0.136	0.182	0.005*	0.252	0.147
HCB									Cells with MN								
CC	−0.017	0.083	0.116	0.079	0.046	0.046	0.045	0.086	CC	0.085	0.065	−0.143	−0.230	−0.153	−0.084	−0.118	0.009
*p*-Value	0.762	0.141	0.039*	0.161	0.41	0.416	0.424	0.127	*p*-Value	0.341	0.464	0.109	0.009*	0.085	0.347	0.185	0.921
*p,p*′-DDE									MN ‰								
CC	0.022	0.049	0.137	0.124	−0.063	0.03	0.104	0.175	CC	0.081	0.038	−0.138	−0.216	−0.147	−0.098	−0.124	0.017
*p*-Value	0.701	0.38	0.014*	0.027*	0.265	0.599	0.063	0.002*	*p*-Value	0.364	0.670	0.122	0.015*	0.098	0.271	0.165	0.851
Cd (blood)									8-OH-dG								
CC	0.058	−0.07	0.113	0.101	0	0.05	0.072	−0.035	CC	−0.004	0.061	0.04	0.044	−0.017	0.022	0.109	0.054
*p*-Value	0.302	0.215	0.045*	0.072	0.996	0.371	0.201	0.534	*p*-Value	0.960	0.447	0.621	0.583	0.831	0.782	0.175	0.504
Cd (urine)									p53								
CC	0.023	0.029	0.118	0.131	0.051	0.103	0.071	−0.085	CC	−0.042	−0.106	−0.111	−0.129	−0.059	−0.153	−0.002	−0.141
*p*-Value	0.676	0.604	0.034*	0.019*	0.363	0.066	0.207	0.13	*p*-Value	0.625	0.214	0.191	0.128	0.490	0.071	0.977	0.096
Pb									CEA								
CC	0.042	0.005	0.013	−0.057	−0.045	−0.105	−0.037	0.003	CC	0.180	−0.119	−0.059	−0.186	−0.008	0.031	−0.094	−0.097
*p*-Value	0.455	0.923	0.819	0.307	0.424	0.061	0.509	0.961	*p*-Value	0.041*	0.178	0.507	0.034*	0.927	0.730	0.286	0.271
*t,t*-MA									Males only								
CC	−0.158	−0.019	−0.056	−0.021	−0.016	−0.056	0.051	−0.086	Dioxins								
*p*-Value	0.007*	0.744	0.341	0.718	0.782	0.334	0.379	0.14	CC	0.012	0.029	0.067	0.054	0.227	0.181	−0.011	0.138
1-OH-pyrene									*p-*Value	0.889	0.729	0.424	0.523	0.006*	0.030*	0.895	0.098
CC	−0.012	0.134	−0.012	0.041	−0.004	0.003	0.147	−0.021	PCBs								
*p*-Value	0.825	0.016*	0.837	0.461	0.946	0.962	0.008*	0.706	CC	0.089	0.092	0.228	0.258	0.150	0.213	0.146	0.233
COMET (median)									*p-*Value	0.262	0.247	0.004*	0.001*	0.058	0.007*	0.065	0.003*
CC	0.096	0.102	0.019	0.095	0.052	0.104	0.049	0.115	HCB								
*p*-Value	0.148	0.123	0.774	0.151	0.431	0.117	0.464	0.082	CC	0.156	0.081	0.225	0.124	0.135	0.062	0.058	0.245
Cells with MN									*p-*Value	0.049*	0.310	0.004*	0.118	0.088	0.438	0.469	0.002*
CC	−0.001	0.093	−0.017	−0.036	−0.017	0.071	−0.002	−0.008	*p,p*′-DDE								
*p*-Value	0.984	0.144	0.785	0.567	0.788	0.261	0.981	0.905	CC	0.109	0.061	0.236	0.193	0.028	0.117	0.105	0.287
MN ‰ ^a^									*p-*Value	0.170	0.444	0.003*	0.015*	0.727	0.141	0.185	0.000*
CC	−0.027	0.075	−0.032	−0.037	−0.009	0.048	−0.022	−0.011	Cd (blood)								
*p*-Value	0.669	0.235	0.616	0.558	0.891	0.452	0.731	0.859	CC	0.114	−0.091	0.133	0.067	−0.015	0.075	0.060	−0.064
8-OH-dG									*p-*Value	0.152	0.255	0.094	0.403	0.853	0.346	0.452	0.426
CC	0.033	−0.008	0.071	0.055	0.013	0.086	0.011	0.031	Cd (urine)								
*p*-Value	0.554	0.884	0.209	0.327	0.811	0.127	0.839	0.579	CC	0.198	0.093	0.224	0.182	0.076	0.205	0.061	0.009
PSA									*p-*Value	0.012*	0.240	0.004*	0.022*	0.337	0.009*	0.440	0.910
CC	−0.115	−0.039	−0.063	−0.111	0.019	0.002	0.073	−0.008	Pb								
*p-*Value	0.146	0.625	0.429	0.164	0.808	0.983	0.358	0.924	CC	0.110	0.049	0.092	0.033	0.020	−0.035	−0.061	0.051
p53									*p-*Value	0.168	0.537	0.247	0.680	0.807	0.665	0.443	0.522
CC	−0.022	−0.044	−0.067	−0.105	−0.084	−0.125	0.02	−0.065	*t,t*-MA								
*p*-Value	0.719	0.459	0.263	0.08	0.161	0.037*	0.734	0.279	CC	−0.115	−0.041	−0.060	−0.057	−0.139	−0.082	0.058	−0.055
CEA									*p-*Value	0.166	0.625	0.470	0.495	0.093	0.321	0.485	0.512
CC	0.113	−0.107	−0.015	−0.097	−0.108	0.018	−0.106	−0.018	1-OH-pyrene								
*p*-Value	0.07	0.085	0.815	0.121	0.082	0.771	0.09	0.779	CC	0.038	0.135	0.029	0.104	−0.026	0.089	0.144	0.029
Females only									*p-*Value	0.633	0.089	0.712	0.189	0.745	0.265	0.069	0.712
Dioxins									COMET (median)								
CC	−0.055	−0.154	−0.086	−0.067	0.056	0.051	−0.129	−0.007	CC	0.039	0.174	−0.055	0.044	−0.037	−0.038	−0.017	0.101
*p*-Value	0.510	0.066	0.307	0.426	0.508	0.542	0.124	0.931	*p-*Value	0.684	0.068	0.566	0.645	0.702	0.692	0.860	0.293
PCBs									Cells with MN								
CC	−0.065	0.008	0.028	0.050	0.013	−0.042	0.145	−0.015	CC	0.003	0.165	0.095	0.089	0.117	0.156	0.140	0.081
*p*-Value	0.413	0.920	0.730	0.531	0.874	0.596	0.068	0.854	*p-*Value	0.973	0.068	0.298	0.326	0.198	0.085	0.123	0.374
HCB									MN ‰								
CC	−0.099	0.122	0.002	−0.044	−0.079	−0.038	0.017	0.041	CC	−0.048	0.157	0.053	0.065	0.128	0.116	0.100	0.069
*p*-Value	0.213	0.124	0.978	0.584	0.325	0.633	0.831	0.611	*p-*Value	0.602	0.083	0.560	0.473	0.158	0.202	0.271	0.449
*p,p*′-DDE									8-OH-dG								
CC	−0.050	0.042	0.028	0.037	−0.163	−0.092	0.099	0.074	CC	0.105	−0.069	0.098	0.040	0.031	0.126	−0.092	0.048
*p*-Value	0.532	0.598	0.723	0.648	0.040*	0.249	0.214	0.354	*p-*Value	0.185	0.383	0.216	0.618	0.696	0.112	0.245	0.544
Cd (blood)									PSA								
CC	0.060	−0.032	0.083	0.092	−0.015	−0.030	0.077	0.084	CC	−0.115	−0.039	−0.063	−0.111	0.019	0.002	0.073	−0.008
*p*-Value	0.452	0.688	0.300	0.251	0.854	0.704	0.334	0.294	*p-*Value	0.146	0.625	0.429	0.164	0.808	0.983	0.358	0.924
Cd (urine)									p53								
CC	−0.062	−0.015	0.005	0.014	−0.011	−0.073	0.070	−0.098	CC	−0.031	0.172	0.020	−0.097	−0.169	−0.122	−0.109	0.101
*p*-Value	0.439	0.849	0.952	0.863	0.886	0.361	0.378	0.220	*p-*Value	0.846	0.281	0.900	0.545	0.290	0.448	0.497	0.530
Pb									CEA								
CC	−0.045	−0.045	−0.065	−0.132	−0.099	−0.178	−0.009	−0.074	CC	0.016	−0.106	0.039	0.028	−0.230	0.018	−0.123	0.041
*p*-Value	0.575	0.572	0.415	0.098	0.216	0.025*	0.910	0.354	*p-*Value	0.859	0.235	0.665	0.754	0.009*	0.838	0.166	0.643
*t,t*-MA																	
CC	−0.164	0.023	−0.055	−0.019	0.093	−0.061	0.036	−0.074									
*p*-Value	0.045*	0.782	0.508	0.815	0.259	0.458	0.662	0.371									

Abbreviations: HCB, hexachlorobenzene; MN ‰, number of micronuclei per 1,000 binucleated cells.

* *p* < 0.05.
